# Gastric cancer cell-derived extracellular vesicles elevate E2F7 expression and activate the MAPK/ERK signaling to promote peritoneal metastasis through the delivery of SNHG12

**DOI:** 10.1038/s41420-022-00925-6

**Published:** 2022-04-05

**Authors:** Fangbin Zhang, Changqing Guo, Xinguang Cao, Yan Yan, Jinping Zhang, Shuai Lv

**Affiliations:** 1 Department of Gastroenterology, The First Affiliated Hospital of Zhengzhou University, 450052 Zhengzhou, P.R. China; 2 Department of Oncology, The First Affiliated Hospital of Zhengzhou University, 450052 Zhengzhou, P.R. China

**Keywords:** Gastric cancer, Gastrointestinal cancer

## Abstract

Cancer cell-derived extracellular vesicles (EVs) have extensive application in the formation of their environment, including metastasis. This study explored the ability of gastric cancer (GC) cell-derived EVs-mediated microRNA-129-5p/E2F transcription factor 7/mitogen-activated protein kinase/extracellular regulated protein kinase (miR-129-5p/E2F7/MAPK/ERK) axis to affect the peritoneal metastasis of GC by delivering lncRNA small nucleolar RNA host gene 12 (SNHG12). EV-derived lncRNA and SNHG12/miR-129-5p/E2F7 network were determined by bioinformatics analysis. The regulatory relationship among SNHG12, miR-129-5p, and E2F7 was verified using a combination of dual-luciferase reporter gene, RNA immunoprecipitation, and RNA pull-down assays. The SNHG12, miR-129-5p, and E2F7 expression was measured by RT-qPCR. After GC cell-derived EVs were isolated and co-cultured with human peritoneal mesothelial cells (HPMCs), the uptake of EVs by HPMCs was observed under laser scanning confocal microscopy. Cell viability and apoptosis were examined using cell counting kit-8 and flow cytometry, respectively. Western blot analysis was performed to measure the mesothelial–mesenchymal transition (MMT)-related protein expression. The pathological and morphological characteristics of metastatic tumors in nude mice were observed by hematoxylin–eosin staining. A high SNHG12 expression was correlated with the poor prognosis of patients with GC. GC-derived EVs led to increased HPMC apoptosis and MMT by transferring SNHG12, whereas the knockdown of SNHG12 annulled the aforementioned results. SNHG12 sponged miR-129-5p to boost E2F7 expression and activate the MAPK/ERK signaling, thus inducing HPMC apoptosis and MMT. In vivo experiments further verified that EVs derived from GC cells promoted peritoneal metastasis in nude mice. GC cell-derived EVs elevated the E2F7 expression and activated the MAPK/ERK signaling to promote peritoneal metastasis through the delivery of SNHG12.

## Introduction

Currently, gastric cancer (GC) has been identified as one leading cause of cancer-associated mortality and one common type of cancer [1]. Peritoneal metastasis is the most prevalent cause of postoperative recurrence in patients with GC [2]. Essentially, an existing infection and a family history of GC in the immediate family are regarded as vital risk factors for GC [3]. Recently, surgical resection has served as the gold-standard therapeutic protocol for early GC, whereas chemotherapy is restricted for the middle and late period irrespective of the recurrent treatment failure due to chemotherapeutic resistance [4]. Notably, extracellular vesicles (EVs) derived from GC cells have elicited function in increasing tumor migration and invasion by regulation of the tumor microenvironment [5].

**Fig. 1:**
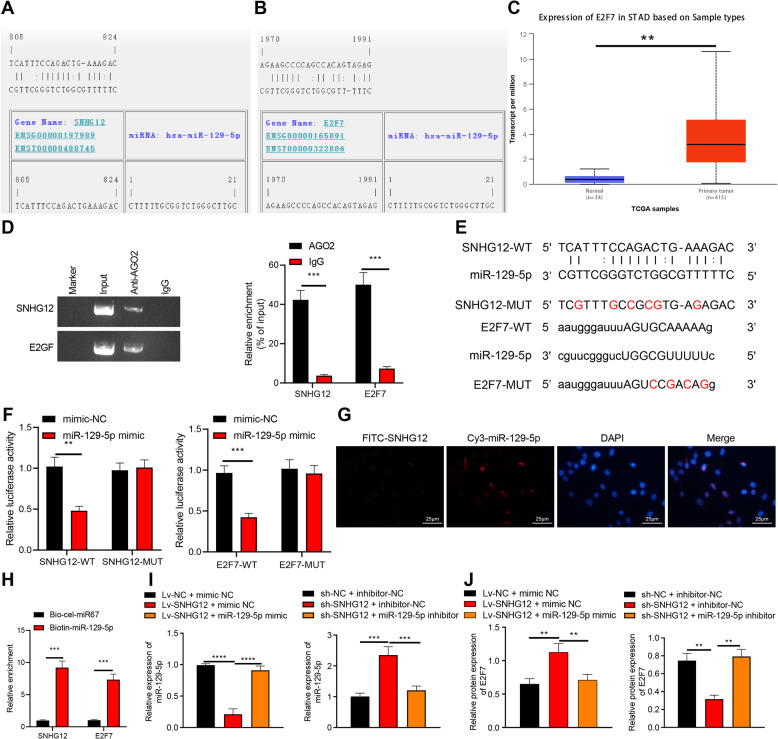
SNHG12 regulates the expression pattern of E2F7 through miRNA-dependent mechanism. **A** The target binding sites of SNHG12 and miR-129-5p predicted by the RNA22 website. **B** The target binding sites of E2F7 and miR-129-5p predicted by the starbase website. **C** The expression box plot of E2F7 in STAD, the gray box represents the expression in normal samples, and the red box the expression in GC samples. **D** Interaction between SNHG12 and E2F7 in HPMCs detected using RIP assay. **E** WT and MUT binding sites between SNHG12 and miR-129-5p as well as between miR-129-5p and E2F7. **F** Luciferase activity analyzed using dual-luciferase reporter gene assay after HEK-293T cells were co-transfected with WT or MUT of SNHG12 or E2F7, miR-129-5p mimic or NC. **G** The co-localization of Cy3-miR-129-5p (red) and FITC-SNHG12 (green) in HPMCs cells measured using FISH (scale bar = 25 μm), and the nucleus was stained with DAPI (blue). **H** Direct binding between SNHG12 or E2F7 and miR-129-5p in HPMCs detected by RNA pull-down assay. **I** miR-129-5p expression pattern in HPMCs evaluated by RT-qPCR. **J** E2F7 protein expression pattern in HPMCs measured by western blot analysis. *p* < 0.05, **p < 0.01, ***p < 0.001, ****p < 0.0001. The experiment was conducted three times independently.

In recent years, EVs have emerged as a significant messenger for cellular communication by the delivery of various bioactive molecules, including proteins, DNA, messenger RNA (mRNA), and non-coding RNA [6]. Existing research work has identified the potential of tumor cell-originated EVs to stimulate mesothelial cell apoptosis, thereby obliterating the peritoneal mesothelial barrier and promoting peritoneal metastasis of GC [7]. LncRNAs in EVs are regarded as novel non-invasive biomarkers for GC [8]. Additionally, lncRNA small nucleolar RNA host gene 12 (SNHG12) is recently identified to elicit pro-carcinogenic effects on GC [9]. An existing study determined an elevated SNHG12 expression in GC tissues with an underlying correlation between the SNHG12 overexpression and the severity of tumor invasion and poor survival [10]. Additionally, upregulated SNHG12 facilitates GC metastasis and epithelial-mesenchymal transformation [11]. Furthermore, SNHG12 can radically modulate microRNA-129-5p (miR-129-5p) via incorporation of the competing endogenous RNA (ceRNA) mechanism [12]. Moreover, miR-129-5p shows an abnormal expression and has a notable functionality in tumor progression; for instance, miR-129-5p is poorly expressed in GC cells [13]. A combination of RNA22- and starbase-based miRNA-mRNA network prediction validated the existence of binding sites between miR-129-5p and E2F transcription factor 7 (E2F7) (Fig. 1A, B). The E2F is a family of transcription factor proteins with extensive functionality, including modulation of cell cycle, cell differentiation, DNA damage responses, and cell death [14]. E2F7 was further analyzed to be overexpressed in GC (Fig. 1C). Additionally, E2F7 regulates the mitogen-activated protein kinase/extracellular regulated protein kinase (MAPK/ERK) [15]. Previously, a notable correlation has been identified between MAPK/ERK and the occurrence and development of GC [16]. In light of the aforementioned literature, we speculated that GC cells released SNHG12-containing EVs to influence peritoneal metastasis of GC through the miR-129-5p/E2F7/MAPK/ERK axis.

## Results

### Highly expressed SNHG12 closely correlates with peritoneal metastasis and poor prognosis in patients with GC

Accumulating evidence has identified that intercellular communication in the GC tumor microenvironment can be transmitted by the regulation of EVs [6, 17]. Thus, we identified 175 significantly upregulated lncRNAs through differential analysis from GC-EVs-related microarray dataset GSE148334, obtained 17,937 human lncRNAs through GENCODE, and selected 21 significantly upregulated lncRNAs in GC-derived EVs through the meticulous intersection (Fig. S1A). Gene Expression Profiling Interactive Analysis (GEPIA) was adopted to analyze significant differences between the 21 lncRNAs provided in the STAD data of The Cancer Genome Atlas (TCGA) database in normal tissues and GC tissues, and we determined that SNHG12, LINC00665, and TTN-AS1 were expressed in GC tissues with the top 3 largest differences (Fig. S1B).

**Fig. 2:**
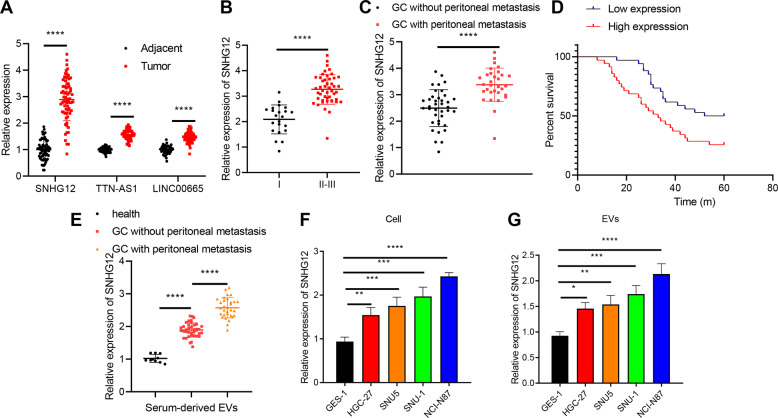
The expression pattern of SNHG12 in GC tissues and cells and its relationship with peritoneal metastasis and prognosis in patients with GC. **A** The expression of SNHG12, LINC00665, and TTN-AS1 in GC and adjacent normal tissues examined using RT-qPCR (*n* = 69). **B** The relative level of SNHG12 in GC tissues from patients with GC of different clinical stages (I (*n* = 22); II and III (*n* = 47)) determined using RT-qPCR. **C** SNHG12 expression pattern in GC tissues with peritoneal metastasis (*n* = 31) and GC tissues without peritoneal metastasis (*n* = 38) from patients with GC detected by RT-qPCR. **D** The correlation between SNHG12 expression pattern and the overall survival of patients with GC analyzed by Kaplan–Meier survival curve. **E** The expression pattern of SNHG12 in serum-derived EVs in 10 healthy volunteers, 38 patients with non-metastatic GC, and 31 patients with metastatic GC determined by RT-qPCR. **F** SNHG12 expression pattern in normal human gastric mucosal cells GES-1 and GC cell lines HGC-27, NCI-N87, SNU-1, and SNU5 measured using RT-qPCR. **G** SNHG12 expression pattern in EVs isolated from each group of GC cells evaluated by RT-qPCR. *p < 0.05, **p < 0.01, ***p < 0.001, ****p < 0.0001. The experiment was conducted three times independently.

Elevated SNHG12, LINC00665, and TTN-AS1 expression in GC tissues was observed relative to the adjacent normal tissues from patients with GC, with a significantly different SNHG12 expression (Fig. 2A). Existing studies have highlighted the correlation between SNHG12 and the development of GC [18, 19]. Additionally, our findings revealed an elevated SNHG12 expression pattern in patients with GC over the pathological staging, while the patients with peritoneal metastasis exhibited a markedly higher SNHG12 expression pattern than those without peritoneal metastasis (Fig. 2B, C). Survival analysis utilizing Kaplan–Meier method suggested an evidently superior prognosis of patients with a low SNHG12 expression relative to those with a high SNHG12 expression (Fig. 2D).

To determine the correlation between EVs-SNHG12 and GC metastasis, we isolated serum-derived EVs from the peripheral blood of healthy volunteers and GC patients with or without peritoneal metastasis. SNHG12 expression was the lowest among the serum-derived EVs of healthy volunteers. However, the SNHG12 expression was elevated in the serum-derived EVs from GC patients with or without peritoneal metastasis, while SNHG12 expression was the highest in GC patients with peritoneal metastasis (Fig. 2E).

To further investigate the role of SNHG12 derived from EVs in vitro, we initially detected the SNHG12 expression pattern in different GC cell lines and isolated EVs. The SNHG12 expression pattern in the GC cell lines (HGC-27, NCI-N87, SNU-1, and SNU5) and derived EVs was evidently elevated compared to the expression pattern in the normal human gastric mucosal cells GES-1 and GES-1-EVs, respectively. Moreover, SNHG12 presented with the highest expression in NCI-N87 cells and NCI-N87-EVs (Fig. 2F, G), which were chosen for subsequent experimentation.

In summary, our findings illustrated a prominent expression pattern of SNHG12 in GC, where the high SNHG12 expression was directly linked to peritoneal metastasis and poor prognosis of GC patients.

### EVs derived from GC cell transfers SNHG12 to HPMCs

**Fig. 3:**
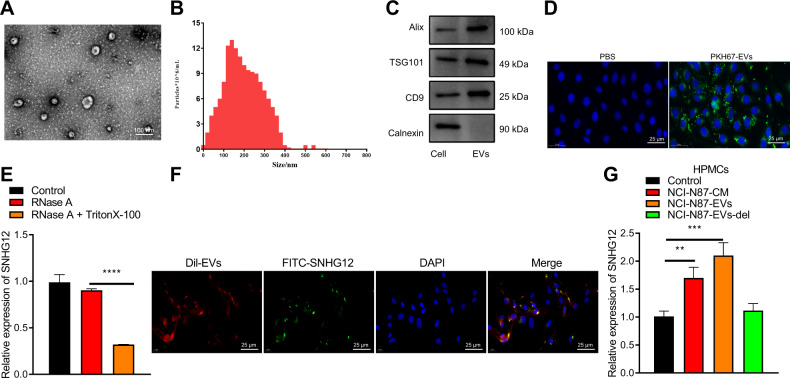
EVs deliver SNHG12 to HPMCs. **A** The morphology of EVs observed using TEM (scale bar = 100 nm). **B** EV diameter distribution measured using NTA. **C** The expression patterns of protein markers of EVs (Alix, TSG101, CD9, and Calnexin) detected by western blot analysis. **D** The admission of NCI-N87-EVs labeled with fluorescent PKH67 into HPMCs (PKH67 labeled EVs were green, DAPI stained nuclei blue, scale bar = 25 μm) observed by confocal laser scanning microscope through *z*-sections and orthogonal views analysis. **E** The expression pattern of SNHG12 after EVs were treated with RNase and Triton X100 detected using RT-qPCR. **F** The enter of FITC-labeled SNHG12 (green) into HPMCs via Dil-labeled EVs (red) observed by confocal laser scanning microscope through *z*-sections and orthogonal views analysis (DAPI: blue, scale bar = 25 µm). **G** The SNHG12 expression pattern in HPMCs detected by RT-qPCR after co-culture of HPMCs and NCI-N87-EVs. *p* < 0.05, **p < 0.01, ***p < 0.001, ****p < 0.0001. The experiment was conducted three times independently.

Transmission electron microscope (TEM) and nanoparticle tracking analysis (NTA) showed that the isolated particles were bilayer vesicles with particle sizes ranging between 30 and 150 nm (Fig. 3A, B). The apoptosis-linked gene-2-interacting protein X (ALIX), tumor suppressor gene 101 (TSG101), and cluster of differentiation 9 (CD9) were positively expressed in EVs, whereas the non-EV marker, Calnexin, was negative (Fig. 3C). To conclude, our results were indicative of the successful isolation of GC cell-derived EVs.

An existing study validated that tumor cell-derived EVs can induce the apoptosis of mesothelial cells, thereby obliterating the peritoneal mesothelial barrier and inducing peritoneal metastasis of GC [7]. In an attempt to determine the terminal effect of SNHG12 from GC-EVs on HPMCs, we isolated and identified HPMCs by immunofluorescence staining (Fig. S2). Our results demonstrated that the staining of Vimentin and Cytokeratin were both positive in HPMCs as the perinuclear cytoplasmic bundles of intermediate filaments. Next, the PKH67-labeled NCI-N87 cell-derived EVs (NCI-N87-EVs) were co-cultured with HPMCs. The results of *z*-sections and orthogonal views detected by confocal laser scanning microscopy illustrated the entrance of a significant concentration of NCI-N87-EVs into HPMCs with distribution around the nucleus (Fig. 3D).

Furthermore, RNase protection experiments were conducted on EVs. After RNase treatment, the SNHG12 expression pattern did not differ significantly. However, the SNHG12 expression pattern was reduced after simultaneous treatment with RNase A and Triton X100, which further indicated that SNHG12 was encapsulated in the membrane rather than released directly (Fig. 3E).

To further explore whether SNHG12 can be transferred in HPMCs via EVs, we transfected fluorescein 5-isothiocyanate (FITC)-SNHG12 (green) into the NCI-N87 cells with subsequent extraction of EVs. As revealed from the z-sections and orthogonal views detected under confocal laser scanning microscopy, the co-localization of FITC and Dil was evident in HPMCs after culture of HPMCs with Dil-labeled EVs (red), thereby suggesting that FITC-SNHG12-containing EVs were internalized by HPMCs (Fig. 3F). Simultaneously, the SNHG12 expression pattern in HPMCs co-cultured with the NCI-N87 cell medium (NCI-N87-CM) or EVs released from NCI-N87 cells was notably higher relative to the negative control (NC). The SNHG12 expression pattern in HPMCs co-cultured with NCI-N87 cells without EVs was not distinctly different (Fig. 3G).

In conclusion, GC cells released EVs transferred SNHG12 to HPMCs.

### GC cells released EVs induce HPMC apoptosis and MMT via delivering SNHG12

**Fig. 4:**
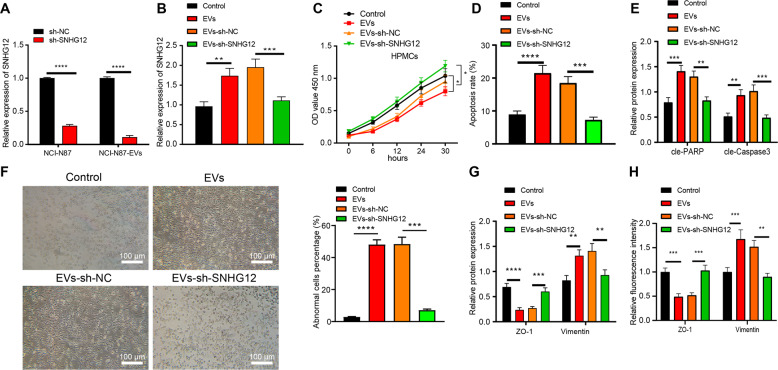
Effects of EVs-SNHG12 from GC cells on apoptosis and MMT of HPMCs. **A** SNHG12 expression pattern in NCI-N87 cells and NCI-N87-EVs measured using RT-qPCR. **B** SNHG12 expression pattern in HPMCs after co-culture detected using RT-qPCR. **C** Cell viability of HPMCs after co-culture monitored using CCK-8. **D** The apoptosis of HPMCs after co-culture examined using flow cytometry. **E** The expression patterns of cleaved PARP and caspase-3 in HPMCs after co-culture determined by western blot analysis. **F** HPMC morphology observed by an inverted microscope (left, scale bar = 100 µm) and the proportion of abnormal cells (right). **G** The expression patterns of ZO-1 and Vimentin in HPMCs after co-culture measured using western blot analysis. **H** The expression patterns of ZO-1 and Vimentin in HPMCs after co-culture measured using immunofluorescence. *p < 0.05, **p < 0.01, ***p < 0.001, ****p < 0.0001. The experiment was conducted three times independently.

As shown in Fig. 4A, SNHG12 expression pattern in NCI-N87 cells and NCI-N87-EVs was downregulated after SNHG12 knockdown. Next, NCI-N87-EVs and HPMCs treated with short hairpin RNA (shRNA) against SNHG12 (sh-SNHG12) were co-incubated and SNHG12 expression in HPMCs treated with EVs-sh-NC or EVs was elevated relative to those untreated. Intriguingly, SNHG12 expression in HPMCs treated with EVs-sh-SNHG12 was decreased (Fig. 4B).

CCK-8 assay and flow cytometry elicited weakened cell viability along with increased apoptosis in HPMCs treated with EVs over time in comparison with those untreated. However, a conflicting trend was evident in HPMCs treated with EVs-sh-SNHG12 (Fig. 4C, D). Moreover, levels of apoptosis-related proteins were examined, results of which expounded that cleaved poly ADP-ribose polymerase (PARP) and caspase-3 expression pattern was elevated in HPMCs treated with EVs compared to those untreated, and their expression pattern was lowered in HPMCs treated with EVs-sh-SNHG12 (Fig. 4E and Fig. S3A).

Then, we evaluated the effect of EVs-SNHG12 on MMT of HPMCs, and our findings revealed the typical polygonal and cobblestone-like appearance of HPMSCs in the control group, while the morphology of HPMCs in the EV group or EVs-sh-NC group significantly altered, similar to the mesothelial cells with increased morphology, while the morphology of HPMCs in the EVs-sh-SNHG12 group was restored (Fig. 4F). Western blot analysis and immunofluorescence showed that Zona occludens 1 (ZO-1) was poorly expressed, whereas Vimentin was highly expressed in HPMCs treated with EVs relative to the control. The ZO-1 expression was amplified whereas Vimentin was downregulated in HPMCs treated with EVs-sh-SNHG12 in comparison with EVs-sh-NC (Fig. 4G, H and Fig. S3B).

Coherently, our findings denoted that EVs released from GC cells induced HPMC apoptosis and MMT by delivering SNHG12.

### SNHG12 can competitively bind to miR-129-5p to increase E2F7 expression

We subsequently sought to investigate the regulatory mechanism underlying the effect of SNHG12 on apoptosis and MMT of HPMCs. As highlighted by accumulating research, SNHG12 can regulate miR-129-5p through the regulation of the ceRNA network [12, 20]. Our findings elicited the presence of sites wherein miR-129-5p bound to SNHG12 and E2F7 through the RNA22 and starbase websites, respectively (Fig. 1A, B). With the help of GEPIA, E2F7 was highly expressed in GC from the STAD data (Fig. 1C). Therefore, we speculated that SNHG12 might work as a potential ceRNA of miR-129-5p to control E2F7.

To validate this hypothesis, we used the Argonaute 2 (AGO2) antibody to perform RNA immunoprecipitation (RIP) assay in HPMCs. SNHG12 and E2F7 were enriched in the AGO2 antibody (Fig. 1D). To further determine whether SNHG12 and E2F7 co-target miR-129-5p, we mutated miR-129-5p binding sites in SNHG12 and E2F7 sequences (Fig. 1E). Dual-luciferase assay demonstrated weakened luciferase activities of SNHG12-wild type (WT) and E2F7-WT in HPMCs manipulated with miR-129-5p mimic compared to mimic-NC (Fig. 1F). Fluorescence in situ hybridization (FISH) results revealed that SNHG12 and miR-129-5p were co-localized in HPMCs (Fig. 1G). Next, based on RNA-pull down experiment, we concluded that SNHG12 and E2F7 were apparently enriched in HPMCs transfected with bio-miR-129-5p (Fig. 1H).

To probe into whether SNHG12 affected E2F7 through miR-129-5p, we overexpressed/silenced SNHG12 and miR-129-5p in HPMCs. miR-129-5p expression pattern was reduced whereas the E2F7 protein expression pattern was increased after overexpression of SNHG12 individually compared to the control group. Additionally, administration of lentivirus harboring SNHG12 (Lv-SNHG12) and miR-129-5p mimic induced an intensified miR-129-5p expression pattern and an inhibited E2F7 protein expression pattern compared to Lv-SNHG12 and mimic-NC. In comparison with the control group, miR-129-5p expression pattern was increased, while the E2F7 expression pattern was lowered after sh-SNHG12 treatment individually in HPMCs. Additionally, co-treatment of sh-SNHG12 and miR-129-5p inhibitor reduced miR-129-5p expression pattern but elevated E2F7 expression pattern in comparison to sh-SNHG12 and inhibitor-NC (Fig. 1I, J).

Conclusively, our findings verified the application of SNHG12 as a definitive ceRNA of miR-129-5p, leading to an increase in miR-129-5p target gene E2F7 expression.

### EVs-SNHG12 promotes E2F7 expression to activate the MAPK/ERK signaling, thus inducing HPMC apoptosis and MMT

We have previously mentioned that EVs derived from GC cells delivered SNHG12 to HPMCs, and SNHG12 regulated E2F7 through modulation of ceRNA-dependent mechanisms. An existing study has validated the ability of E2F7 to mediate the MAPK/ERK signaling [15]. Numerous studies have determined a correlation between MAPK/ERK and the development of GC [16, 21]. Hence, we explored whether EVs-SNHG12 derived from GC cells induced HPMC injury via the miR-129-5p/E2F7/MAPK/ERK axis.

**Fig. 5:**
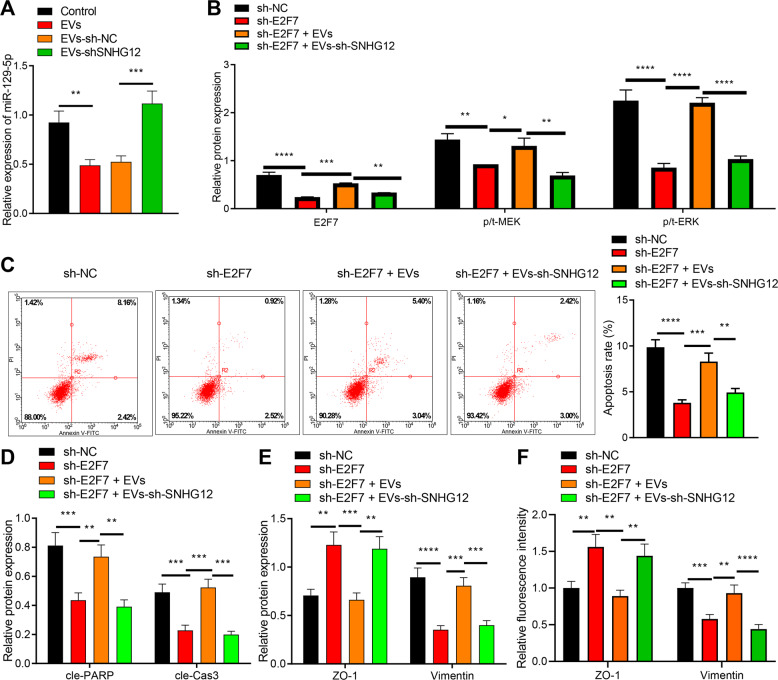
EVs-SNHG12 regulates the miR-129-5p/E2F7/MAPK/ERK axis to affect HPMC apoptosis and MMT. **A** miR-129-5p expression pattern in HPMCs treated with EVs measured using RT-qPCR. **B** The expression patterns of E2F7, p/t-MEK, and p/t-ERK in HPMCs treated with EVs detected using western blot analysis. **C** Apoptosis of HPMCs after co-culture examined using flow cytometry. **D** The expression patterns of cleaved PARP and caspase-3 in HPMCs after co-culture examined by western blot analysis. **E** The expression patterns of ZO-1 and Vimentin in HPMCs after co-culture determined using western blot analysis. **F** The expression patterns of ZO-1 and Vimentin in HPMCs after co-culture determined using immunofluorescence. *p < 0.05, **p < 0.01, ***p < 0.001, ****p < 0.0001. The experiment was conducted three times independently.

Initially, the miR-129-5p expression pattern after HPMCs co-cultured with GC cell-derived EVs was evaluated. The administration of EVs induced a notably downregulated miR-129-5p expression pattern compared with the control. miR-129-5p expression pattern was intensified after EVs-sh-SNHG12 treatment in comparison with those treated with EVs-sh-NC (Fig. 5A). After HPMCs transfected with sh-E2F7 were co-cultured with GC cells released EVs, our findings denoted that the E2F7 expression pattern and phosphorylated/total MEK (p/t-MEK) as well as p/t-ERK expression were all suppressed after sh-E2F7 treatment. However, the E2F7 expression pattern, p/t-MEK and p/t-ERK in HPMCs transfected with sh-E2F7 and EVs was elevated. Moreover, the administration of sh-E2F7 and EVs-sh-SNHG12 led to notable downregulation of E2F7, p/t-MEK, and p/t-ERK (Fig. 5B and Fig. S3C).

We further studied the effect on HPMC apoptosis and MMT. sh-E2F7 transfected HPMCs exhibited lowered apoptosis and MMT compared to sh-NC transfected HPMCs. However, administration of sh-E2F7 and EVs resulted in enhanced apoptosis and MMT. Furthermore, significantly suppressed HPMC apoptosis and MMT were detected in HPMCs after sh-E2F7 and EVs-sh-SNHG12 treatment (Fig. 5C–F and Fig. S3D).

Subsequently, EVs derived from GC cells delivered SNHG12 to HPMCs by competitively adsorbing miR-129-5p to increase the E2F7 expression pattern, thus initiating MAPK/ERK signaling to subsequently facilitate HPMC apoptosis and MMT.

### GC cells released EVs-SNHG12 promotes metastasis in vivo via miR-129-5p/E2F7/MAPK/ERK axis

**Fig. 6:**
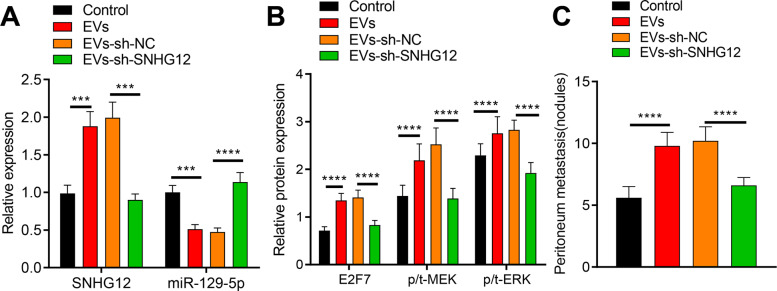
GC cells derived EVs-SNHG12 affects peritoneal metastasis of GC cells. Note: NCI-N87 cells were injected alone or co-injected with EVs. **A** The expression patterns of SNHG12 and miR-129-5p in peritoneal tissues of nude mice evaluated using RT-qPCR. **B** The expression patterns of E2F7, p/t-MEK, and p/t-ERK in peritoneal tissues of nude mice evaluated using western blot analysis. **C** The number of peritoneal nodules in nude mice detected by HE staining. *n* = 15. *p* < 0.05, *p* < 0.01, ***p < 0.001, ****p < 0.0001. The experiment was conducted three times independently.

EVs treatment elevated expression patterns of SNHG12, E2F7, p/t-MEK, and p/t-ERK but depleted miR-129-5p expression pattern compared to the control group. Additionally, SNHG12, E2F7, p/t-MEK, and p/t-ERK expression pattern in tissues was reduced prominently whereas miR-129-5p expression pattern was increased when tumor-bearing mice were treated with EVs-sh-SNHG12 in comparison to EVs-sh-NC (Fig. 6A, B and Fig. S3E).

Meanwhile, the metastatic peritoneal nodules in tissues treated with EVs were increased compared to the control group as demonstrated by hematoxylin–eosin (HE) staining. However, the metastatic peritoneal nodules in tissues of tumor-bearing mice treated with EVs-sh-SNHG12 were reduced in comparison with EVs-sh-NC (Fig. 6C).

EVs derived from GC cells harboring SNHG12 promoted the peritoneal metastasis of GC cells via miR-129-5p/E2F7/MAPK/ERK axis.

## Discussion

GC is a heterogeneous disease characterized by alterations in epidemiology and histopathology depending on the region and is the primary cause of cancer-associated mortality worldwide [22]. Due to the underlying limitation of an established national routine screening program, the majority of GC cases in China are classified in an advanced stage upon diagnosis [23]. For locally advanced disease, a combination of adjuvant or neoadjuvant therapy with surgery is recommended, however for the patients with metastatic disease and poor prognosis, the median survival rate is merely about 1 year [24]. Essentially, peritoneal metastasis is regarded as the chief reason for the poor prognosis of patients with advanced GC, and accumulating evidence has demonstrated the potential of cancer cell-derived EVs in body fluids to aid the diagnosis and treatment of GC [25]. Furthermore, lncRNAs have elicited notable functionality in tumor growth, metastasis, invasion, and prognosis in GC by regulation of the tumor microenvironment in EVs [17]. LncRNAs released from EVs have now been identified as definitive bio-markers for GC [26]. The current study sought to investigate the vital role of EV-derived SNHG12 released from GC cells in GC. Consequently, our findings elicited that GC cell-derived EVs elevated E2F7 expression and activated the MAPK/ERK signaling to induce peritoneal metastasis of GC by the delivery of SNHG12.

Initially, we revealed elevated SNHG12 expression in GC and overexpressed SNHG12 was closely associated with peritoneal metastasis and poor prognosis of GC patients. The SNHG12 expression was evidently upregulated in GC tissues, which negatively correlated with the overall survival time [18]. Additionally, SNHG12 was strikingly elevated in GC, and its expression was closely linked with the tumor size, tumor lymph node metastasis stage, distant metastasis, and lymphatic metastasis [27]. Moreover, our results illustrated that GC cell-derived EVs delivered SNHG12 to HPMCs as evidenced by the elevated SNHG12 expression in NCI-N87-derived EVs co-cultured HPMCs. Similarly, an existing study identified a notable elevated EVs-derived HOXA distal transcript antisense RNA is in GC [28]. In addition, overexpression of lncRNA zinc fnger antisense 1 was detected in serum delivered by EVs in patients with GC [29]. Next, EVs derived from GC cells induced HPMC apoptosis and MMT by delivering SNHG12, which denoted that EVs-SNHG12 HPMCs exhibited increased apoptosis, reduced activity, and a silenced ZO-1 expression but boosted Vimentin and caspase −3 levels. In consistent with our findings, existing research revealed that SPOD1-AS embedded in ovarian cancer-secreted EVs was delivered to mesothelial cells to induce MMT and facilitate peritoneal colonization [30]. Moreover, overexpression of COX-2 has been evident in peritoneal adhesions and the inhibition of COX-2 can improve the prognosis of lipopolysaccharide-induced HMPC injury, promote cell viability, inhibit cell apoptosis, and production of inflammatory cytokines [31]. Moreover, elevated expression levels of Vimentin along with down-regulated ZO-1 mRNA and protein expression in patients with peritoneal fibrosis [32]. Additionally, our findings elicited that EVs derived from GC cells transferred SNHG12 to induce HPMC apoptosis and MMT.

The following experiment demonstrated the capacity of SNHG12 to bind to miR-129-5p to upregulate E2F7. An existing study identified an inverse relation between SNHG12 and miR-129-5p and further speculated that SNHG12 could serve as a metabolic sponge of miR-129-5p [20]. miR-129-5p negatively targets E2F7 [33]. Notably, SNHG12 can function as a sponge for miR-129-5p to increase the MAPK1 and E2F7 expression [34]. Subsequently, our findings denoted that EVs-SNHG12 promoted the E2F7 expression to activate the MAPK/ERK signaling to induce HPMC injury. In consistent with our study, an existing study elicited an overexpression of E2F7 in colorectal cancer cells and tissues where it could mediate MAPK/ERK signaling [15]. Overexpression of lncRNA breast cancer antiestrogen resistance 4 activates the MAPK/ERK signaling, thereby exercising a carcinogenic effect in GC [35]. Additionally, our results revealed that SNHG7 and E2F7 were upregulated, while miR-181a-5p is underexpressed in non-small-cell lung cancer. Moreover, inhibition of E2F7 induces suppressed cell viability as well as enhanced cell apoptosis [36]. Altogether, our study verified that EVs-SNHG12 derived from GC cells promoted metastasis of GC cells in vivo.

**Fig. 7:**
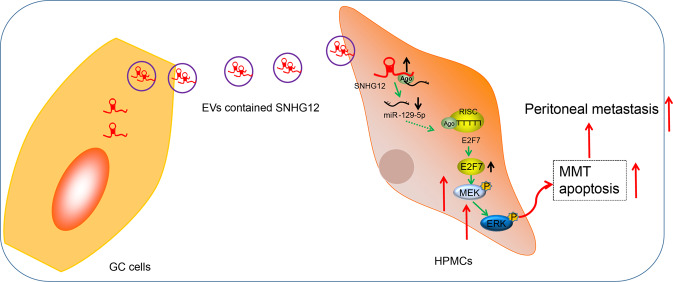
The molecular mechanism of gastric cancer (GC) cell-derived extracellular vesicles (EVs) in peritoneal metastasis of GC. Note: gastric cancer cell-derived extracellular vesicles elevate E2F7 expression and activate the MAPK/ERK signaling to promote peritoneal metastasis through the delivery of SNHG12. SNHG12 lncRNA small nucleolar RNA host gene 12, miR-129-5p microRNA-129-5p, E2F E2F transcription factor 7, MAPK mitogen-activated protein kinase, ERK extracellular regulated protein kinase.

To conclude, GC cells released EVs manipulated the miR-129-5p/E2F7 axis by transferring SNHG12 to HPMCs to activate the MAPK/ERK signaling, thereby ultimately increasing GC peritoneal metastasis (Fig. 7). The current study principally focused on the role of EVs-derived SNHG12 from GC cells in GC, which offers an innovative and prospective target for the therapy of GC. Further studies are warranted. Looking forward, progressive steps in the study of the further elucidation and targeted prevention of GC are expected to be expounded.

## Materials and methods

### Bioinformatics methods

GC-derived EV-related GSE148334 database was obtained through the Gene Expression Omnibus database, from which five samples were identified, including one normal EVs sample and four GC-derived EV samples. Differential analysis was conducted using the GEO2R tool, and log >3 and *p* < 0.05 were set as the screening threshold. The name of human lncRNA was retrieved from GENCODE. The STAD data in the TGCA database were analyzed by analysis of existing literature and using GEPIA to obtain the respective expression of genes in GC. The target and binding sites of lncRNA and miRNA were predicted using the RNA22 website, while downstream targets of miRNA were predicted and the binding site was obtained using starbase.

### Collection of tissue samples

Tissue and blood samples were sourced from patients with GC and healthy volunteers at the First Affiliated Hospital of Zhengzhou University. 69 matched GC and adjacent normal tissues were collected. Among the participants, there were 40 males and 29 females with a mean age of 62.14 ± 7.11 years ranging from 48 to 73 years. Thirty-one cases of GC with peritoneal metastasis and 38 cases of GC without peritoneal metastasis were documented. According to Enneking’s pathological staging, 22 cases were classified in stage I, 16 cases in stage II, and 31 cases in stage III. The patients with GC were initially diagnosed with histopathological evaluation in the First Affiliated Hospital of Zhengzhou University, and all patients did not receive any treatment before the biopsy. The tissue specimens were immediately preserved at −80 °C. The abdominal omentum was harvested from patients who underwent abdominal surgery.

### Cell culture

GC cell line human gastric mucosal cell line (GES-1) was provided by Procell (Wuhan, Hubei, China) and the GC cell lines (HGC-27, NCI-N87, and SNU-1) were supplied by the Cell Bank of the Chinese Academy of Sciences (Shanghai, China). The GC cell line SNU5 and HEK-293T were provided by ATCC (Manassas, VA, USA). The cell lines were preserved in RPMI-1640 medium (Gibco Laboratories, Grand Island, NY, USA) containing a combination of 10% (v/v) fetal bovine serum (FBS, Sigma-Aldrich, St. Louis, USA), 100 IU/mL penicillin (Sigma-Aldrich), and 100 mg/mL streptomycin (Sigma-Aldrich) for culture at 37 °C with 5% CO_2_.

Isolation and purification of HPMCs were as follows: 10 mm^2^ of abdominal omentum was harvested, cut into pieces, and rinsed for 5 min × 3 times with phosphate-buffered solution (PBS) under aseptic conditions. Next, the HPMCs were detached using 0.1% type I collagenase at 37 °C for 2 h and centrifuged at 1200 × *g* for 10 min to eliminate the upper layer of undigested adipose tissues and oil, resuspended, filtered through a 200-mesh sieve, followed by repeated centrifugation. Next, the red blood cell lysis buffer was added for lysis of HPMCs for 5 min. Lastly, cells were resuspended in RPMI-1640 medium harboring 10% FBS and inoculated into 6 cm^2^ culture plates at 5 × 10^4^ cells/cm^2^. The cells inoculated for the first time were considered as passage 0. Upon attaining 90% confluence, 0.25% trypsin was added for subsequent treatment. The cells were passaged at the proportion of 1:3 and the cells at passages 2 and 3 were selected. Immunofluorescent staining was performed with the corresponding mouse monoclonal antibodies against Cytokeratin (ab52625, 1:100, Abcam, Cambridge, UK) and Vimentin (ab8978, 1:100, Abcam) to identify HPMCs.

### Immunofluorescent staining

Cells were seeded on a 24-well plate at 1 × 10^5^ cells/well. After 2 days, cells were fixed using pre-chilled 4% paraformaldehyde (C104190, Aladdin Biochemical Technology, Shanghai, China) for 20 min. Then, 0.1% of TritonX-100 (T109026, Aladdin) was added for incubation for 15 min to increase the permeability. Subsequently, cells were incubated in 1% bovine serum albumin (BSA) for 30 min for blockade of any non-specific binding sites. Next, an incubation lasted for 1 h with the use of primary antibodies against Cytokeratin (ab52625, 1:100, Abcam, ZO-1 (ab61357, 1:250, Abcam), and Vimentin (ab8978, 1:100, Abcam). Afterward, secondary antibody Alexa Fluor 568-conjugated goat anti-mouse immunoglobulin G (IgG) (ab175473, 1: 500, Abcam) or AlexaFluor 488-conjugated goat anti-rabbit IgG (ab150077, 1:200, Abcam) was added for incubation for 1 h. Next, Hoechst 33342 (1:10,000; 1 mg/mL) was added for nuclear staining. The stained cells were imaged under a fluorescence microscope.

### Cell transfection

The HEK-293T cells were treated with plasmids expressing pLenti-SNHG12, Lv-SNHG12, Lv-NC, pLenti-sh-SNHG12, sh-SNHG12, sh-NC, pLenti-E2F7 or pLenti-sh-E2F7 using the lentivirus packaging kit (Invitrogen, Carlsbad, CA, USA). The final concentration of shRNA was 100 nM. The virus supernatant was collected after 48 h, and the virus concentration was evaluated by Genechem (Shanghai, China). The HPMCs and GC cells were subsequently cultured to about 50% cell confluence, then infected with lentivirus (1 × 10^8^ TU/mL), respectively, screened with 10 μg/mL puromycin (Sigma-Aldrich) 48 h after infection and maintained for 1 week for a selection of stably transfected cell lines.

miR-129-5p mimic, miR-129-5p inhibitor, and corresponding NC were provided by GeneChem. HPMCs were cultured in a 6-well plate cell culture dish at 4 × 10^5^ cells/mL. The cells of 80% confluence were transfected using lipofectamine 2000 (11668-019, Invitrogen).

### Isolation and purification of EVs

Isolation and purification of the GC cell lines-derived EVs was as follows: FBS was ultracentrifuged in advance and centrifuged at 100,000 × *g* for 18 h to eliminate EVs. The GC cell lines were cultured in RPMI-1640 medium containing 10% FBS to attain 80–90% confluence. The culture medium was then replaced with 10% FBS medium with EVs for subsequent culture in an incubator at 37 °C with CO_2_ for 48 h. The supernatant harvested above was gradient-centrifuged (500 × *g* for 15 min, 2000 × *g* for 15 min, and 10,000 × *g* for 20 min) to eliminate any cell debris, apoptotic bodies, or large vesicles. After filtration with the 0.22-micron filter, cells were centrifuged at 110,000 × *g* for 70 min at 4 °C, and then resuspended using PBS under the same conditions. Next, cells were resuspended with 100 μL sterile PBS, followed by quantification and diameter measurement using NTA, RNA extraction, protein extraction, as well as cell and animal processing. All ultracentrifugation was conducted at 4 °C with Beckman UltracentRifuge (Optima L-90K, USA) equipped with SW-32Ti rotors. The remaining low-speed centrifuges were performed using the Beckman Allegra X-15R tabletop centrifuge.

Additionally, the serum-derived EVs were separated and purified using the Capturem™ Extracellular Vesicle Isolation Kit (635741, Takara, Beijing, China).

### Identification of EVs

Nanoparticle particle size analysis was as follows: 20 μg EVs were dissolved in 1 mL PBS and mixed for 1 min for homogenous distribution. The NTA tool (Malvern, UK) was adopted for the particle size distribution of EVs.

TEM observation was as follows: 20 μL of ultra-isolated fresh EVs was loaded onto a carbon-coated copper electron microscope grid for 2 min, and then dyed in phosphotungstate solution (12501-23-4, Sigma-Aldrich) for 5 min. The grid was rinsed by PBS to eliminate any excess phosphotungstate solution, and semi-dried. The observation image was captured at 80 KV under a Hitachi H7650 TEM (Japan).

Western blot analysis was conducted for the identification of the EV-surface markers. After the concentration of EV suspension, the bicinchoninic acid (BCA) kit (23227, Thermo Fisher Scientific, San Jose, CA, USA) was utilized for protein concentration measurement. Next, a regimen of sodium dodecyl sulfate-polyacrylamide gel was prepared and the protein denaturation and electrophoresis were performed. Subsequently, the proteins on gels were transferred onto the membrane for analysis of the EV-specific proteins TSG101 (ab125011), CD9 (ab263019), Alix (ab388388), and Calnexin (ab22595) with antibodies from Abcam.

### Verification of SNHG12 loaded into EVs

RNase A treatment was adopted to examine whether the lncRNA was surface-bound or packaged in EVs. Briefly, EVs were resuspended, and 20 μg/μL RNase A (Purelink RNase A, Life technologies, Waltham, MA, USA) was added for incubation at 37 °C for 20 min. The integrity of the EV membrane was compromised with a cleaning agent (TritonX-100), and the radioimmunoprecipitation assay (RIPA) buffer was added for a 20-min reaction, followed by the aforementioned RNase A treatment. After incubated with RNase A, the lysate buffer was added to inhibit the reaction and isolate the RNA content. The SNHG12 expression was determined.

### Cellular uptake of EVs

Purified GC cell-derived EVs were tagged by PKH67 with a kit from Sigma-Aldrich. The EVs were resuspended in 1 mL of Diluent C solution, and 4 μL PKH67 ethanol dye solution was mixed with 1 mL of Diluent C solution to prepare 4 × 10^−6^ M dye solution. Then, 1 mL EV suspension was dyed in the preceding dye solution for 5 min, which was terminated with 2 mL of 1% EVs-depleted FBS for 1 min. The PKH67-tagged EVs were ultracentrifuged at 100,000 × *g* for 2 h, and the EVs in the samples were enriched in the sucrose density range of 1.13–1.19 g/mL, and subsequently harvested. PKH67-tagged EVs were incubated with the GC cells at 37 °C for 12 h. The nuclei were tagged utilizing 4,6-diamino-2-phenylindole (DAPI; D9542, Sigma-Aldrich). Finally, a fluorescence microscope (ECLIPSE E800, Nikon, Japan) was employed for observing the uptake of EVs by GC cells.

To identify the transfer of SNHG12, FITC was added to label SNHG12 in the GC cells. The cell supernatant was collected, and the EVs were separated in compliance with the aforementioned centrifugation steps. The dil label (red) reagent was added for incubation with HPMCs for 48 h. The nuclei of HPMCs were stained with DAPI (D9542, Sigma-Aldrich). A confocal laser scanning microscope (LSM 710, Carl Zeiss, Thornwood, NY, USA) was adopted for observation of the co-localization of FITC and Dil in HPMCs through *z*-sections and orthogonal views analysis [37].

### Cell morphological observation

The HPMCs were treated with EVs, EVs-sh-NC, and EVs-sh-SNHG12, followed by cellular morphology observation under a microscope (Confocal Microscope Leica TCS SP8 X, Germany). The proportion of abnormal cells was evaluated under 10 different fields with at least 500 cells according to the following formula: the proportion of abnormal cells = the number of abnormal cells/the number of total cells × 100%.

### RT-qPCR

The total RNA content was extracted using Trizol (Cat. No.16096020, Thermo Fisher Scientific), and 5 µg was reserved for the synthesis of cDNA using the RT kit (K1622, Fermentas, Burlington, Ontario, CA, USA). Real-time PCR of mRNA was performed using SYBR Premix Ex Taq kit (Takara) and ABI StepOne real-time PCR system (Applied Biosystems, Foster City, CA, USA). Real-time PCR of miRNA was performed using the miRcute Plus miRNA qPCR assay kit (Tiangen Biotech). mRNA expression was normalized to glyceraldehyde-phosphate dehydrogenase (GAPDH) and exogenous cel-miR-39, while miRNA expression was normalized to U6. The miRNA universal reverse primer was provided by miRcute Plus miRNA First-Strand cDNA Synthesis Kit (Tiangen Biotech), and the other was synthesized by Sangon (Table S1). The target gene relative expression was estimated based on 2^−ΔΔCT^ method.

### Identification of miRNA-mRNA and miRNA-lncRNA relations

SNHG12 sequence containing miR-129-5p binding site and the mutation binding site was cloned into the pGL3-luciferase reporter vector (Promega, Madison, WI, USA) to construct the SNHG12-WT and SNHG12-MUT reporter vectors. Next, HEK-293T cells were co-introduced with miR-129-5p mimic/mimic NC and the afore-mentioned reporter vectors with the assistance of Lipofectamine 2000 (Invitrogen).

The same method was used to detect the binding ability of miR-129-5p and E2F7. A combination of miR-129-5p mimic or mimic NC with luciferase reporter plasmid pmirGLO dual-luciferase miRNA Target Expression vector (E1330, Promega) containing WT E2F7-3’untranslated region (UTR) or MUT E2F7-3’UTR were co-transfected into the HEK293T cells.

The dual-luciferase reporter analysis system (E1910, Promega) was utilized for luciferase activity detection.

### RIP assay

Magna-RNA binding protein IP kit (Millipore, Billerica, MA, USA) was tested. RIP buffer carrying magnetic beads pre-bound with human anti-AGO2, or normal nude mouse IgG as a NC was added to the whole-cell lysate for incubation with the addition of proteinase K samples. Furthermore, the immunoprecipitated RNA was subsequently isolated. The concentration was measured using a spectrophotometer (Thermo Fisher Scientific) and the quality of RNA was determined by the bio-analyzer (Agilent, Santa Clara, CA, USA). Lastly, the RNA content was extracted and the purified RNA content was subject to analysis by quantitative real-time PCR to identify the presence of binding targets.

### RNA pull down

HPMCs were transfected with biotin-labeled RNA, biotin-labeled miR-129-5p (bio-miR-129-5p) and NC (bio-cel-miR67) (50 nM each). After 48 h of transfection, the cells were isolated and lysed for 10 min with a specific buffer (Ambion). Next, the cell lysate was subpackaged (50 mL each). The remaining lysate was incubated with the M-280 streptavidin magnetic beads (Sigma-Aldrich) pre-coated with RNase-free and yeast tRNA (Sigma-Aldrich) for 3 h at 4 °C. Next, the cells were rinsed twice with the cold lysis buffer, three times with low-salt buffer, and once with the high-salt buffer. The RNA content was extracted before RT-qPCR determination of SNHG12 or E2F7 expression in bio-miR-129-5p pull-down samples.

### FISH

Cells of 60–70% confluence were fixed and permeabilized with 0.5% TritonX-100 for 15 min at 4 °C. The FITC-labeled SNHG12 cell probes were incubated at 37 °C overnight, and then rinsed in 2× saline- sodium citrate (SSC) 6 times for 3 min. Next, the Cy3-marked miR-129-5p probes and pre-hybridization buffer (at a dilution ratio of 1:100) were incubated for 3–5 min at 88 °C. Following incubation together with the Cy3-marked miR-129-5p probes at 37 °C overnight, the cells were rinsed 6 times in 2× SSC for 3 min. Following DAPI dying, the cells were rinsed 3 times for 5 min in 2× SSC. Five non-overlapping visual fields were chosen for observation and documentation of the findings under a fluorescence microscope (Olympus, Japan).

### Flow cytometric apoptotic analysis

Cells were isolated using trypsin treatment. The cells were then harvested following centrifugation. The cell density was adjusted to 10^6^ cells/mL. Subsequently, 200 μL of cells were centrifuged, resuspended in 100 μL binding buffer, supplemented with 2 μL of Annexin V-FITC (20 μg/mL) (Life Technologies, V13241), allowed to rest on ice for 15 min in conditions devoid of light, and then transferred to the flow tube. After the addition of 300 μL PBS, each sample was supplemented with 1 μL propidium iodide (PI) (50 μg/mL). The examination was performed within 30 min.

### CCK-8 assay

The cells were seeded in a 96-well plate at a concentration of 3 × 10^4^ cells/mL (100 μL/well) and 15 wells were inoculated. Transfection was conducted after culture for 24 h. Detection proceeded at 0, 6, 12, 24, and 30 h after transfection. After analysis, 10 μL CCK-8 (Sigma-Aldrich) was added to each well. After continuous incubation for 1 h in the cell incubator, the absorbance at 450 nm was measured using a microplate meter (NYW-96M, Nuoyawei Instrument & Meter, Beijing, China). The cell viability curve was plotted with the time point as the abscissa and optical density value as the ordinate.

### Western blot analysis

High-efficiency RIPA lysis (R0010, Solarbio, Beijing, China) was employed to extract total protein. After quantification, the protein electrophoresed on polyacrylamide gel was then loaded onto a polyvinylidene fluoride membrane, which was then subject to blockade using 5% BSA for 1 h. The membrane was probed with corresponding primary rabbit antibodies at 4 °C in a shaker overnight: E2F7 (NBP1-80266, 1:1000, Bio-Techne), p-MEK (ab96379, 1:500), MEK (ab32576, 1:500), p-ERK (ab201015, 1:500), ERK (ab184699, 1:500), cle-PARP (ab32064, 1:1000), cle-caspase3 (ab32042, 1:500), ZO-1 (ab96587, 1:1000), Vimentin (ab92547, 1:1000), and GAPDH (ab8245, 1:10,000). The aforementioned antibodies except E2F7 were provided by Abcam. The membrane was then incubated with horseradish peroxidase-marked goat anti-rabbit IgG (AB_2819160, 1:20,000, Abcam) for 1 h and supplemented with the developer solution for development. ImageJ 1.48 software (National Institutes of Health, Bethesda, MD, USA) served as a tool for grayscale analysis.

### Tumorigenicity assay in vivo

A total of 60, 4–5-week-old male immunodeficiency nude mice (BALB/c, nu/nu; Charles River Laboratories, Beijing, China) were bred under non-pathogenic conditions at 26–28 °C with 50–65% saturated humidity. In the nude mice peritoneal metastasis model, 5 × 10^6^ GC cells were injected into the nude mice via tail vein, and drug administration was performed every 5 days. The mice were randomly divided into 4 groups with 15 mice in each group. The mice were respectively injected with PBS, 10 μg of GC cell-derived EVs, GC cell-derived EVs infected with a lentiviral vector expressing shRNA against NC (EV sh-NC), or GC cell-derived EVs infected with sh-SNHG12. After 20 days, all nude mice were euthanized with CO_2_, and the mesenteric tissues were isolated for later use. The investigator was blinded to the group allocation during the experiment and when assessing the outcome.

### HE staining

The mesenteric saline was fixed in 4% paraformaldehyde for 30–50 min, dehydrated, transparentized, paraffin-immersed, embedded, and finally sliced. The tissue sections were flattened and pasted on the slides, dried in a thermostatic chamber at 45 °C, then dewaxed with xylene, and rinsed under distilled water for 2 min through high to low concentration alcohol. The sections were dyed in hematoxylin solution for 5 min, reacted in 1% hydrochloric acid and ethanol for 3 sec, dyed using 5% eosin solution for approximately 2 min, then dehydrated, transparentized, and mounted. The tissue sections were visualized under a microscope.

### Statistical analysis

All data were analyzed using GraphPad Prism 8.0. The measurement data were represented as mean ± standard deviation. Paired *t* test was conducted for two group comparisons of experimental data that conformed to normal distribution and homogeneity of variance, while unpaired *t* test was utilized for comparing the unpaired data. One-way analysis of variance (ANOVA) was applied for multiple group comparisons, followed by Tukey’s post hoc test. Two-way ANOVA or repeated-measures ANOVA was used for multi-group comparison at multiple time points, followed by Tukey’s post hoc test. Log-rank method was used for the statistical test of Kaplan–Meier survival curves. In all statistical references, experimental values of *p* < 0.05, *p* < 0.01, *p* < 0.001, and *p* < 0.0001 were indicative of a statistically significant difference.

## Supplementary information

Supplemental Material Files

## Data Availability

All data generated or analyzed during this study are included in this article and/or its Supplemental Material files. Further inquiries can be directed to the corresponding author.
