# Supplementary material for: Gastric cancer cell-derived extracellular vesicles elevate E2F7 expression and activate the MAPK/ERK signaling to promote peritoneal metastasis through the delivery of SNHG12
Source: Cell Death Discov. 2022 Apr 5;8:164. doi: 10.1038/s41420-022-00925-6 (PMC8983762; doi:10.1038/s41420-022-00925-6)
Supplement: Supplementary file 1 — Supplemental Material Files [file 41420_2022_925_MOESM1_ESM.docx]

**Supplemental Material Files**

**Figure S1** The significantly upregulated lncRNA obtained using bioinformatics analysis. A, Venn map of significantly upregulated lncRNA in GC cell-derived EVs obtained from GSE148334 microarray. B, Expression box plot of SNHG12, LINC00665 and TTN-AS1 in STAD data. **p* < 0.05.


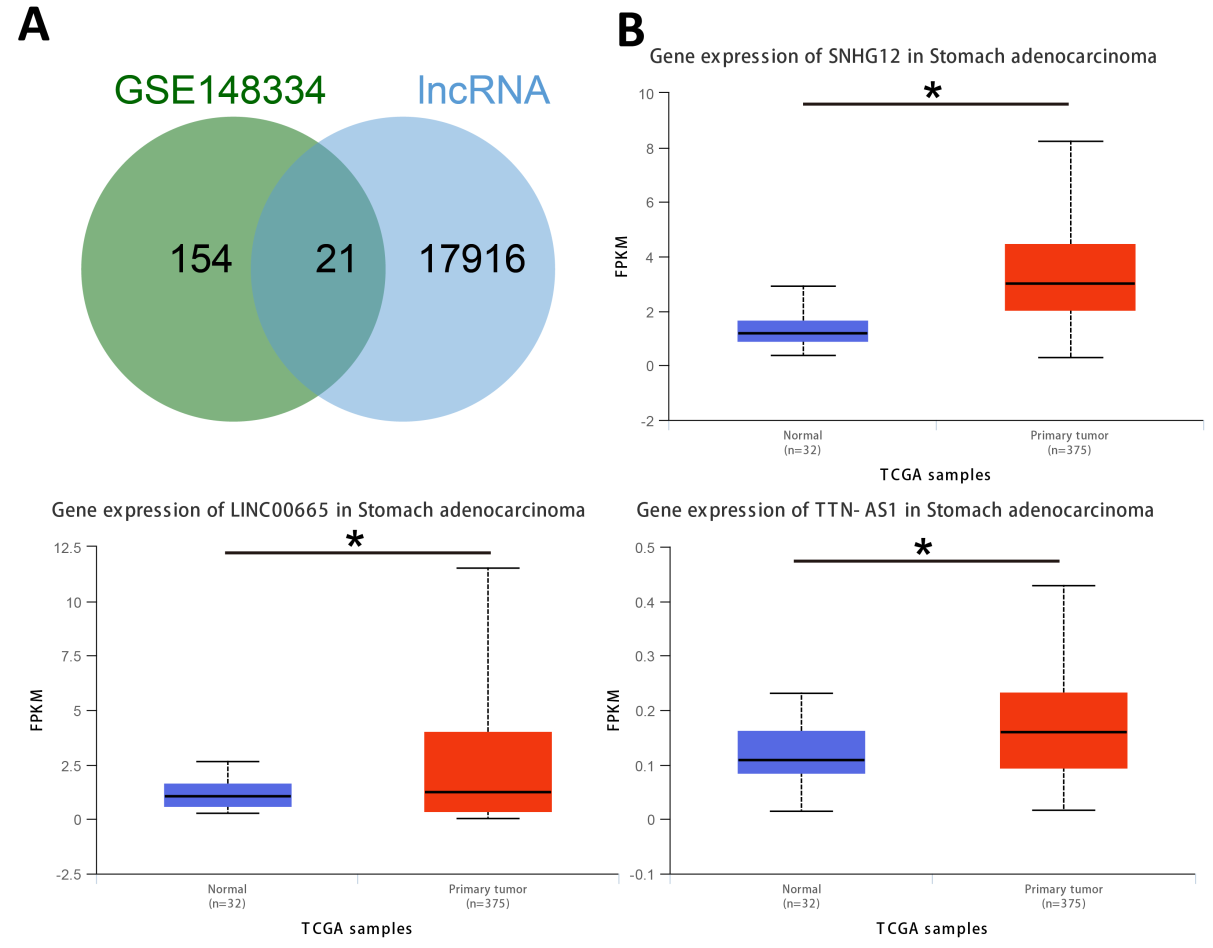


**Figure S2** The expression patterns of Vimentin and cytokeratin in HPMCs was identified using immunofluorescence.


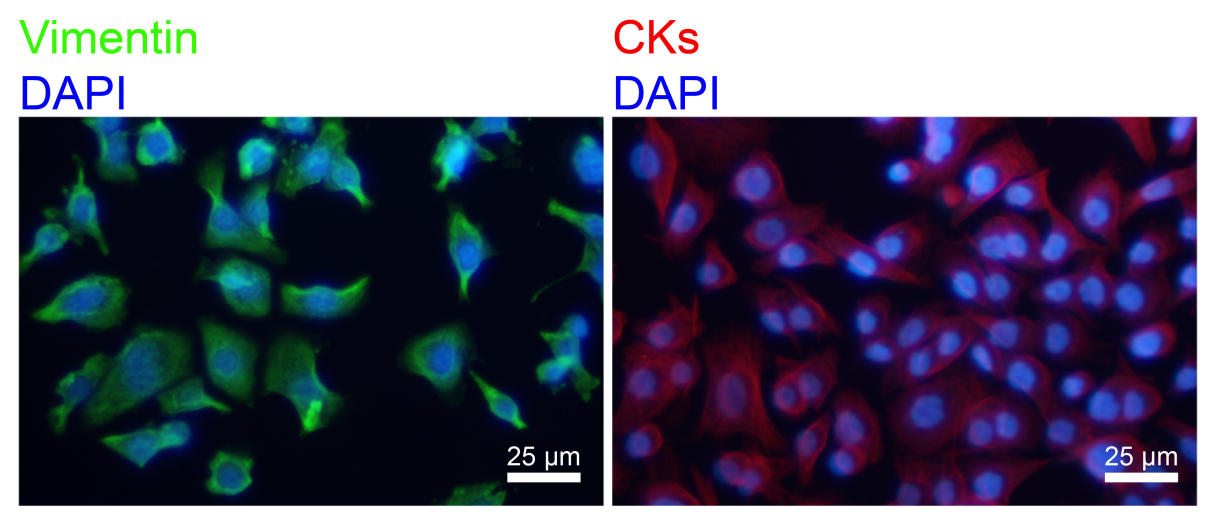


**Figure S3** The original western blots of Figure 3E (A), 3G (B), 5B (C), 5D (D), and 6B (E).

**
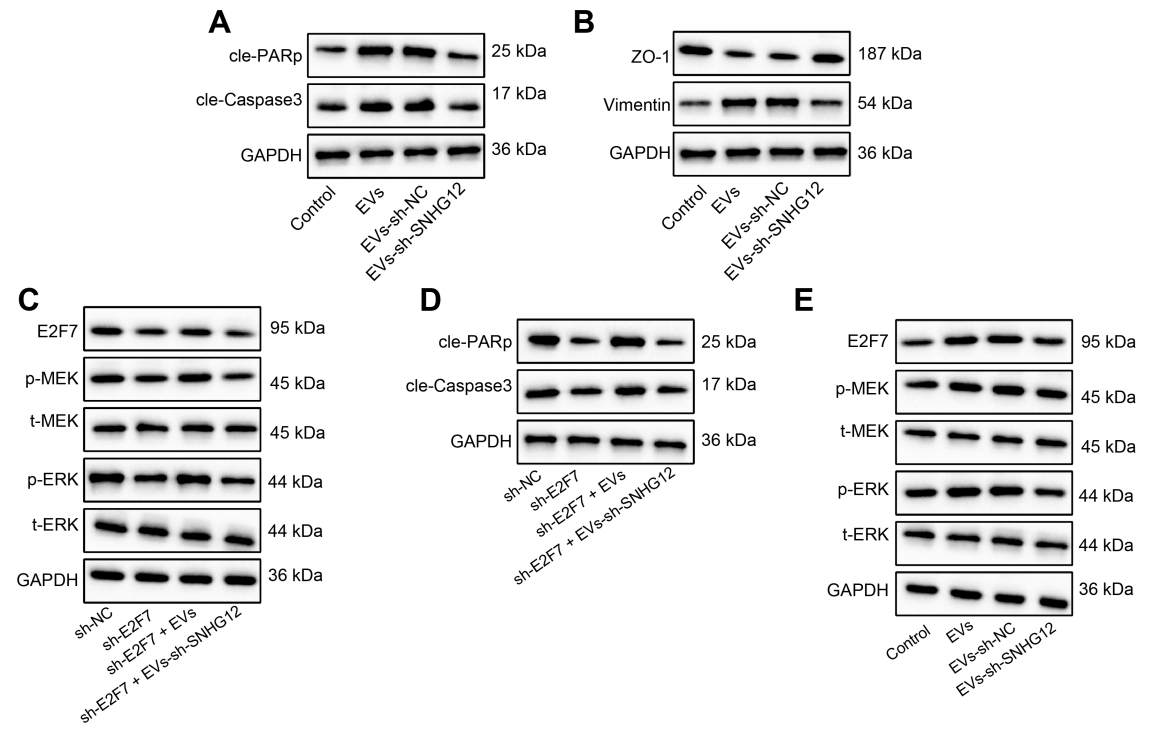
**

**Table S1** RT-qPCR primer sequences

| Targets | Primer sequences (5' - 3') | |
| --- | --- | --- |
| miR-129-5p | F: TGCGGTCTGGGCTTGC | Universal reverse primers |
| cel-miR-39 | F: CGTCTTGGTAATAGTCGTATCCA | Universal reverse primers |
| U6 | F: CTCGCTTCGGCAGCACA | Universal reverse primers |
| SNHG12 | F: TGACAGGCGGATAAAACGGT | R: AGTACGCCGGGATCTCTGTA |
| E2F7 | F: CTGCTGCGCTAGACTTGGAT | R: TCTCTTAGTAGGACCACCAACG |
| GAPDH | F: AGAAGGCTGGGGCTCATTTG | R: AGGGGCCATCCACAGTCTTC |

Note: miR-129-5p, microRNA-129-5p; SNHG12, small nucleolar RNA host gene 12; U6, small nuclear RNA; E2F7, E2F transcription factor 7; GAPDH, glyceraldehyde-3-phosphate dehydrogenase; RT-qPCR, reverse transcription quantitative polymerase chain reaction; F, forward; R, reverse.
